# Assessing alcohol intake & its dose-dependent effects on liver enzymes by 24-h recall and questionnaire using NHANES 2001-2010 data

**DOI:** 10.1186/s12937-016-0180-y

**Published:** 2016-06-22

**Authors:** Sanjiv Agarwal, Victor L. Fulgoni, Harris R. Lieberman

**Affiliations:** 1 Oak Ridge Institute for Science and Education, Belcamp, MD 21017 USA; 2 Henry M. Jackson Foundation, Bethesda, MD 20817 USA; 3 Military Nutrition Division, U.S. Army Research Institute of Environmental Medicine, Natick, MA 01760-5007 USA

**Keywords:** Alkaline phosphatase, Alanine aminotransferase, Aspartate aminotransferase, Gamma glutamyl transferase, Bilirubin, NCI method

## Abstract

**Background:**

Alcohol is a significant component of the diet with dose-dependent risks and benefits. High doses of alcohol damage the liver and early symptoms of liver disease include changes in routinely assessed liver enzymes. Less is known regarding the mechanisms responsible for the benefits of moderate alcohol consumption, including their effects on the liver. The objectives of this study were to examine alcohol’s dose-dependent effects on markers of liver function (alkaline phosphatase (ALP), alanine aminotransferase (ALT), aspartate aminotransferase (AST), gamma glutamyl transferase (GGT), and bilirubin), as well as to compare the different methods of assessing alcohol intake using NHANES 2001–2010 adult data (*N* = 24,807).

**Methods:**

Three methods were used to estimate alcohol intake from all volunteers: 24-h recall; the National Cancer Institute (NCI) method of usual intake; and a specific alcohol intake questionnaire.

**Results:**

Mean alcohol intake by 24-h recall, NCI method and questionnaire was 41.0 ± 0.8 g/d, 10.9 ± 0.2 g/d and 11.0 ± 0.2 g/d, respectively. Alcohol consumers had significantly lower levels of ALP and higher levels of AST, GGT and bilirubin compared to non-consumers (*P* < 0.01) and activities of ALT, AST, and GGT increased and of ALP decreased as alcohol intake increased, regardless of intake assessment method used. The most sensitive measure of alcohol consumption was GGT.

**Conclusions:**

Since alcohol had a graded linear effect on several liver enzymes, including at low and moderate doses, benefits as well as risks of alcohol intake may be related to liver function. Since the NCI method and alcohol questionnaire yielded very similar alcohol intake estimates, this study cross-validated these methods and demonstrated the robustness of the NCI method for estimating intake of irregularly consumed foods.

## Introduction

Alcohol is consumed by about 70 % of U.S. adults [1]. Consumption of this dietary component has both risks and benefits. Low to moderate doses of alcohol lower the risk of cardiovascular disease and all-cause mortality [2]. The Dietary Guidelines for Americans 2010 and American Heart Association’s dietary guidelines suggest that if alcohol is consumed, males consume no more than two alcoholic drinks per/day (28 g/day) and women no more than one per/day (14 g/day) [2, 3]. However, approximately 38 million adults in the United States report binge drinking an average of four times per month and consuming an average of eight drinks per episode [4]. Excessive alcohol intake was responsible for approximately 10 % of deaths among working age adults in the United States during 2006–2010 [5] and cost the United States $223.5 billion in 2006 [6]. It is estimated to be the fourth leading preventable cause of death in the United States [7]. Heavy drinking, including binge drinking, increases the risk of liver disease, hypertension, stroke, type II diabetes, gastrointestinal cancers, injuries and violence [2]. Alcohol abuse is the leading cause of liver-related morbidity and mortality [8]. In alcoholic patients increased levels of several liver-derived biomarkers are associated with excessive ethanol intake and alcoholic liver disease, and a number of studies have reported induction of liver enzyme function due to excessive alcohol consumption [9–17]. However, only a few small studies have investigated the effects of moderate alcohol consumption on liver enzymes [12, 13].

The National Health and Nutrition Examination Survey (NHANES), a large nationally representative survey of the U.S. population, is designed to monitor the health and nutritional status of adults and children [18]. The NHANES data are currently released every 2 years with data from approximately 5000 adult volunteers and include an in-home survey and physical examination conducted in a mobile facility. Food consumption, body composition, health status, multiple blood chemistry measures, and various other health-related parameters are assessed. Because the NHANES is conducted regularly using the same standardized procedures, very large data sets can be obtained by combining multiple years of data. This provides an opportunity to examine, in a large nationally-representative data set, self-reported alcohol use and its association with various markers of liver function. In addition, the NHANES employs two methods that can be used to independently estimate alcohol, collection of two days of 24-h recall data which then can be used to estimate usual intake and an alcohol intake questionnaire.

The primary objective of this study was to examine the effects of graded levels of alcohol intake on markers of liver function using the very large, nationally representative, NHANES data set. We also compared several methods for assessing alcohol intake: 1) single 24-h recall; 2) usual alcohol intake based on the two 24-h recalls collected by NHANES and adjusted using the National Cancer Institute (NCI) usual intake procedure; and 3) an alcohol consumption questionnaire.

## Methods

### Study population

NHANES data are collected by the National Center for Health Statistics (NCHS) of the Center for Disease Control and Prevention (CDC) on a nationally representative sample of non-institutionalized Americans [18]. All participants or proxies provided written informed consent and the Research Ethics Review Board at the NCHS approved the survey protocol. Data from NHANES 2001–2002, 2003–2004, 2005–2006, 2007–2008 and 2009–2010 were combined for the analysis. The combined sample included 24,807 adults age 19 years and older (12,561 males and 12,246 females) and excluded pregnant or lactating females, those with incomplete dietary records or missing liver enzyme data. Response rates for NHANES are typically quite good, but they are related to age; for example medical examination response rated typically exceed 80 % for persons under age 20 but tend to be less than 70 % for those age 70 or more [19].

### Estimates of alcohol intake

Alcohol intake (g/day) was assessed three ways: 1) using a single 24-h recall, an estimate of an individual’s self-reported alcohol consumption on the day of recall; 2) by determining usual alcohol intakes with the NCI method, an estimate of long-term intake that employed data from 2 days of 24-h recalls; and 3) via an alcohol intake questionnaire quantifying annual consumption of alcohol. Participants completed an in-person 24-h dietary recall and health examination in a Mobile Examination Center and a second 24-h dietary recall was collected via telephone 3–10 day after the first recall using the United States Department of Agriculture’s automated multiple-pass method. A detailed description of the survey design and the data collection procedures are available elsewhere [18]. The usual intake analysis used the NCI method with a two part correlated model in which the probability of non-zero intake on a given day and the usual intake on consumption days were estimated and assumed to be correlated [20]. The two-part correlated model was used since unlike nutrient intake, alcohol is not consumed on most days by most people. The MIXTRAN and INDIVNT macros were used to estimate individual usual intakes with a recall day and weekday/weekend (Friday-Sunday) intake indicator in the model. Additionally, since an alcohol questionnaire was administered during the NHANES household interview it was also used to assess alcohol intake as average number of drinks per day over the last year [18]. The alcohol content of alcoholic drinks is defined as 12 oz of beer, 5 oz of wine or 1.5 oz of liquor and equals 14 g of alcohol [8, 21].

### Outcome variables for markers of liver function

As part of the NHANES in-person health examination in the Mobile Examination Center, participants provided a blood specimen for laboratory analyses. Fasting prior to collection of the blood sample was not required. The activities of alkaline phosphatase (ALP), alanine aminotransferase (ALT), aspartate aminotransferase (AST) and gamma glutamyl transferase (GGT) were measured spectrophotometerically in U/L units using their respective kinetic enzymatic methods, and bilirubin levels in mg/dl were measured spectrophotometerically using a timed-endpoint Diazo method [18]. ALT and AST are aminotransferases that, when elevated, are indicative of reduced hepatocyte integrity. GGT and bilirubin are markers of cholestasis [18, 22–24] and elevated levels of bilirubin are associated with hemolytic jaundice [18, 22–24].

### Statistical analysis

Analyses were conducted using SAS 9.2 (SAS Institute, Inc.; Cary, NC) and SUDAAN release 11.0 (Research Triangle Institute, Research Triangle Park, NC). Appropriate weighting factors were used to adjust for oversampling of selected groups, non-response to the survey by some individuals, and day of the week when the interview was conducted. For each alcohol assessment approach, subjects were grouped into deciles of intake as well as one of seven alcohol consumption groups in g/d: 0, 0 to ≤7, >7 to ≤14, >14 to ≤21, >21 to ≤28, >28 to ≤35, and >35 (the usual intake method did not have a 0 g/d group). These levels are approximately equivalent to 0, 0 to ≤0.5, >0.5 to ≤1.0, >1.0 to ≤1.5, >1.5 to ≤2.0, >2.0 to ≤2.5 and >2.5 drinks/d, respectively. Least square (LS) means and standard errors (SE) of enzyme activities were determined using PROC REGRESS of SUDAAN after adjustment for various covariates. Outcome variables were adjusted for race-ethnicity, age, physical activity (categorized as sedentary, moderate, or vigorous based on responses to questions on activity), poverty income ratio, smoking habits (yes/no), energy intake, and BMI. Laboratory test values for ALP, ALT, AST, GGT, and bilirubin were compared across deciles of alcohol intake as well as alcohol intake levels in g/day and effects of alcohol intake on liver enzymes were assessed to ascertain whether an increase in alcohol intake was associated with changes in enzyme activities and whether changes were linear or curvilinear as a function of alcohol. We used the Dunnett significant difference post-hoc test (DUNNETT test option within PROC GLM) to compare each category of alcohol intake to the non/low alcohol consumer group. These post-hoc tests were sample weighted but not adjusted for the complex sample design of NHANES as this capability was not available.

While not the primary focus of this research, we did examine the impact of age, gender, socioeconomic status (measured as poverty income ratio) and presence of hypertension (based on the answer to a question about whether a subject had previously been told by a doctor they had hypertension or elevated blood pressure) on liver markers. We also examined the interaction of alcohol intake (as measured by all three methods of assessment) with age, gender, race/ethnicity, socioeconomic status, and presence of hypertension. These regression models also contained additional covariates: physical activity (categorized as sedentary, moderate, or vigorous based on response to questions on activity), current smoking (yes/no), energy intake, and BMI. Significance for all statistical comparisons was set at *P* < 0.01.

## Results

Approximately 58.7 % of NHANES 2001–2010 adult participants (*n* = 13,104) regularly consumed alcohol (questionnaire method) and 25 % of adult participants consumed alcohol (*n* = 6176) at least once over the 24-h survey period (24-h recall method). Regardless of the method used to identify alcohol consumers, they were more likely to be male, younger, non-Hispanic whites, smokers, have higher income, and have more physically-active lifestyles compared to non-consumers.

The mean intake of alcohol as estimated by the 24-h recall method representing the alcohol intake on the day of recall was about 3–5 times higher than the usual intake estimated by NCI method or by alcohol intake questionnaire (Table 1). Usual intake, as assessed by the NCI method using two 24-h recalls, and intake by questionnaire representing long term intake, provided very similar estimates of alcohol consumption.

**Table 1:** Average intake of alcohol by three different methods of estimation^a^, NHANES 2001–2010

Mean alcohol intake g/day		Gender combined	Male	Female
24-h Recall	*N*	*24,807*	*12,561*	*12,246*
	Mean ± SE	41.0 ± 0.8	48.2 ± 1.0	29.4 ± 0.8
Usual intake by NCI method	*N*	*24,807*	*12,561*	*12,246*
	Mean ± SE	10.9 ± 0.2	16.1 ± 0.3	5.9 ± 0.1
Alcohol intake questionnaire	*N*	*22,307*	*11,376*	*10,931*
	Mean ± SE	11.0 ± 0.2	14.6 ± 0.3	6.4 ± 0.2

(*N *Sample Size; *SE* Standard Error; ^a^Alcohol intake (g/day) was assessed three ways: 1) via a single 24-h recall; 2) by determining usual alcohol intakes with the NCI method, an estimate of long-term intake that employed data from two days of 24-h recalls; and 3) via an alcohol intake questionnaire quantifying annual consumption of alcohol)

**Fig. 1:**
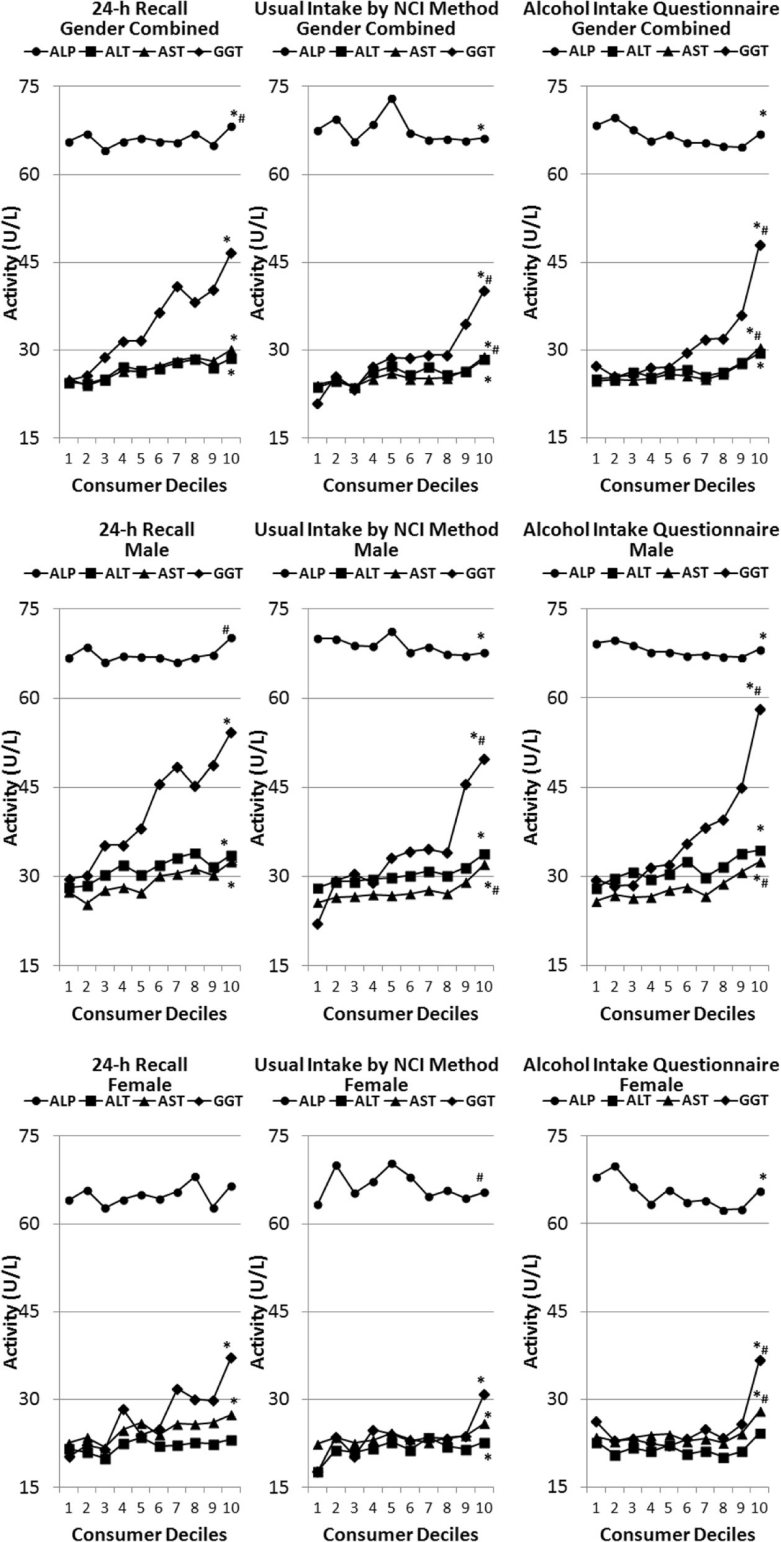
Association of alcohol consumption by consumer deciles of intake with liver enzyme function† NHANES 2001–2010. † *ALP* alkaline phosphatase, *ALT* alanine aminotransferase, *AST* aspartate aminotransferase and *GGT* gamma glutamyl transferase; * Significant Linear Trend at *P* < 0.01; ^#^ Significant Curvilinear Trend at *P* < 0.01

**Fig. 2:**
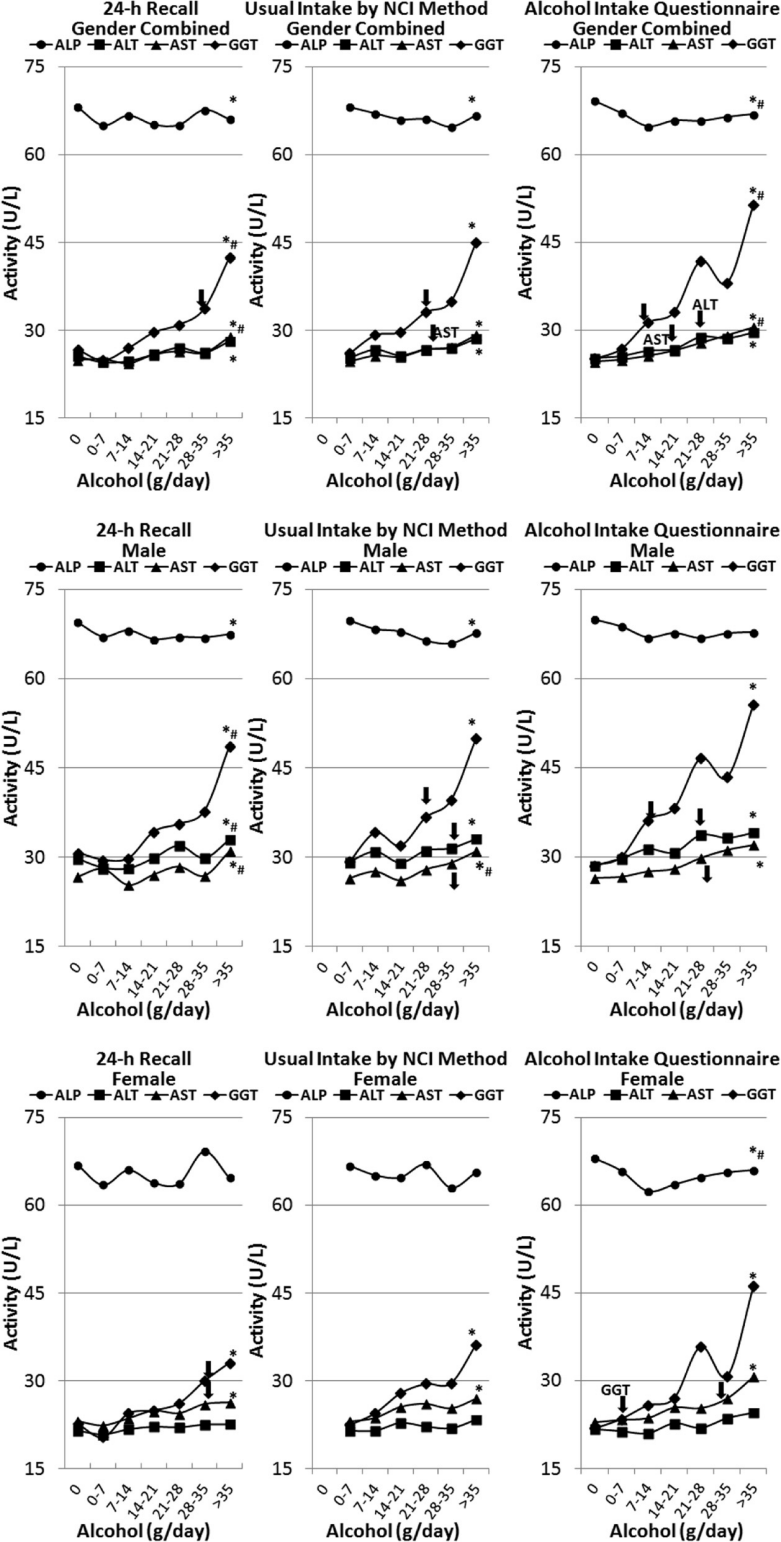
Association of alcohol consumption by g of alcohol per day with liver enzyme function† NHANES 2001–2010. † *ALP* alkaline phosphatase, *ALT* alanine aminotransferase, *AST* aspartate aminotransferase and *GGT* gamma glutamyl transferase; * Significant Linear Trend at *P* < 0.01; #Significant Curvilinear Trend at *P* < 0.01; ↓Significant Dunnett at *P* < 0.01 continuous start point

The activities of liver enzymes of alcohol consumers and non-consumers were compared and alcohol consumers (gender combined) had significantly lower ALP (5–6 %, *P* < 0.01) and higher AST (4–7 %, *P* < 0.01), GGT (15–25 %, *P* < 0.01) and bilirubin (3 %, *P* < 0.01) compared to non-consumers by both methods. However, the difference in ALT activities of alcohol consumers and non-consumers, were less than 3 % and were not statistically significant (*P* > 0.01). To determine whether there were dose-related differences in liver enzymes levels in alcohol users, we examined the data by deciles of alcohol intake (Fig. 1) as well as alcohol intake by g/day (Fig. 2), estimated by three different methods. Table 2 provides the percentile of amount of alcohol intake by consumers using different methods of alcohol intake estimation and can be used to assess the percentage of the population consuming various levels of alcohol. Regardless of method used, a significant increasing linear trend in activities of ALT, AST, and GGT, and a decreasing linear trend in activity of ALP, was observed as alcohol intake increased (Figs. 1 and 2, Table 3). A significant curvilinear trend was noted for activity of ALP, AST and GGT with some methods but not with others (Figs. 1 and 2, Table 3) indicating modification of the direction and/or magnitude of the change as alcohol intake increases. While the relationship between intake of alcohol and activities of AST and GGT was similar in males and females, the relationship between alcohol intake and activities of ALP and ALT were largely driven by males (Figs. 1 and 2, Tables 4 and 5). The increase in activity of GGT with increase in alcohol intake was higher than the increase in activities of ALT and AST, regardless of the method used to measure alcohol intake (Figs. 1 and 2, Tables 3, 4 and 5). However, no significant trends were noted for bilirubin levels (Tables 3, 4 and 5). Dunnett post-hoc test indicated that, based on the alcohol questionnaire data, we could detect a significant difference in GGT with consumption of as little as 7–14 g alcohol per day (*P* < 0.01) while differences were detected for AST and ALT at 14–21 and 21–28 g alcohol per day, respectively (*P* < 0.01).

**Table 2:** Percentiles of amount of alcohol intake by consumers estimated by three different methods of alcohol intake estimation among adults. NHANES 2001–2010

	Alcohol intake (g/day ± SE) percentiles				
	10th	25th	50th	75th	90th
24-h recall					
All	4.52 ± 1.01	13.96 ± 0.02	28.05 ± 0.03	55.60 ± 1.56	88.50 ± 2.16
Male	10.93 ± 0.56	15.59 ± 0.85	33.48 ± 0.97	63.07 ± 2.30	102.9 ± 4.02
Female	0.74 ± 0.16	11.04 ± 0.40	21.21 ± 0.53	36.95 ± 1.21	64.71 ± 2.56
Usual intake by NCI method					
All	1.84 ± 0.003	2.60 ± 0.003	5.30 ± 0.08	7.15 ± 0.49	30.20 ± 0.65
Male	3.32 ± 0.17	5.34 ± 0.01	6.70 ± 0.11	20.38 ± 0.74	42.66 ± 1.00
Female	1.38 ± 0.01	1.99 ± 0.05	2.61 ± 0.002	3.47 ± 0.002	15.94 ± 0.70
Alcohol intake questionnaire					
All	0.23 ± 0.02	0.92 ± 0.002	4.00 ± 0.001	13.96 ± 0.50	27.89 ± 0.05
Male	0.44 ± 0.02	1.85 ± 0.06	7.98 ± 0.25	19.96 ± 0.28	39.42 ± 0.60
Female	0.16 ± 0.02	0.46 ± 0.001	2.00 ± 0.20	7.84 ± 0.07	15.95 ± 1.12

*SE* Standard Error; An alcoholic drink is defined as equals 14 g of alcohol [8, 21]

**Table 3:** Association of liver enzyme function with alcohol consumption level (Deciles and g/day) by three different methods of alcohol intake estimation among all adults. NHANES 2001–2010^a^

		24-h recall	Usual intake by NCI method	Alcohol intake questionnaire
		Regression coefficient^b^		
Alkaline phosphatase (ALP), U/L				
	*N*	*21,245*	*21,245*	*19,523*
By Decile	Linear	47.82/−0.24*	50.70/−0.38*	48.37/−0.45*
	Curvilinear	48.14/−1.10/0.11^#^	ns	ns
By g/day	Linear	48.21/−0.37*	48.70/−0.45*	47.58/−0.53*
	Curvilinear	ns	ns	51.08/−3.14/0.36^#^
Alanine aminotransferase (ALT), U/L				
	*N*	*21,179*	*21,179*	*19,460*
By Decile	Linear	9.95/0.26*	7.17/0.37*	9.22/0.27*
	Curvilinear	ns	ns	ns
By g/day	Linear	9.59/0.34*	8.84/0.57*	8.91/0.74*
	Curvilinear	ns	ns	ns
Aspartate aminotransferase (AST), U/L				
	*N*	*21,177*	*21,177*	*19,458*
By Decile	Linear	18.57/0.42*	15.58/0.40*	17.06/0.33*
	Curvilinear	ns	17.82/−0.36/0.06^#^	17.75/−0.35/0.08^#^
By g/day	Linear	18.00/0.54*	17.01/0.79*	16.64/0.93*
	Curvilinear	19.28/−0.70/0.17^#^	ns	17.86/0.02/0.12^#^
Gamma glutamyl transferase (GGT), U/L				
	*N*	*21,245*	*21,245*	*19,523*
By Decile	Linear	−0.64/1.65*	−13.55/1.72*	−5.78/1.43*
	Curvilinear	ns	−8.30/−0.05/0.15^#^	−3.01/−1.30/0.31^#^
By g/day	Linear	−2.94/2.21*	−7.09/3.29*	−7.36/3.88*
	Curvilinear	2.31/−2.95/0.69^#^	ns	−3.21/0.80/0.42^#^
Bilirubin (mg/dl)				
	*N*	*21,236*	*21,236*	*19,514*
By Decile	Linear	0.87/0.003*	0.86/0.001	0.84/0.005*
	Curvilinear	ns	ns	ns
By g/day	Linear	0.87/0.004*	0.86/0.004	0.85/0.01*
	Curvilinear	ns	ns	ns

^a^Regression analyses examining linear and/or quadratic effect of alcohol intake conducted with the following covariates: age, gender, race/ethnicity, current smoking status (yes/no), poverty income ratio, physical activity level (sedentary, moderate, or vigorous based on feedback on questions on activity), energy intake, and body mass index. ^b^β_0_/β_1_ for Linear Trend and β_0_/β_1_/β_2_ for Curvilinear Trend (β_0_: intercept, β_1_: linear regression coefficient, and β_2_: curvilinear regression coefficient); * Significant Linear Trend at *P* < 0.01; ^#^ Significant Curvilinear Trend at *P* < 0.01, *N* Sample Size; *ns* Non-significant Curvilinear Trend

**Table 4:** Association of liver enzyme function with alcohol consumption level (Deciles and g/day) by three different methods of alcohol intake estimation among male adults. NHANES 2001–2010^a^

		24-h recall	Usual intake by NCI method	Alcohol intake questionnaire
		Regression coefficient^b^		
Alkaline phosphatase (ALP), U/L				
	*N*	*10,858*	*10,858*	*10,025*
By Decile	Linear	66.64/−0.25	68.96/−0.37*	65.82/−0.29*
	Curvilinear	67.25/−1.24/0.12^#^	ns	ns
By g/day	Linear	67.20/−0.40*	68.07/−0.53*	65.67/−0.40
	Curvilinear	ns	ns	ns
Alanine aminotransferase (ALT), U/L				
	*N*	*10,820*	*10,820*	*9987*
By Decile	Linear	15.08/0.36*	12.14/0.47*	14.24/0.52*
	Curvilinear	ns	ns	ns
By g/day	Linear	14.46/0.45*	13.25/0.68*	13.94/0.93*
	Curvilinear	16.10/−1.14/0.21^#^	ns	ns
Aspartate aminotransferase (AST), U/L				
	*N*	*10,819*	*10,819*	*9986*
By Decile	Linear	22.45/0.48*	19.35/0.50*	21.06/0.45*
	Curvilinear	ns	23.03/−0.75/0.11^#^	21.92/−0.35/0.09^#^
By g/day	Linear	21.66/0.57*	20.30/0.80*	20.47/0.94*
	Curvilinear	23.50/−1.22/0.23^#^	23.07/−0.75/0.18^#^	ns
Gamma glutamyl transferase (GGT), U/L				
	*N*	*10,858*	*10,858*	*10,025*
By Decile	Linear	7.97/2.12*	−6.88/2.38*	0.44/2.14*
	Curvilinear	ns	3.35/−1.07/0.30^#^	4.23/−1.37/0.39^#^
By g/day	Linear	4.36/2.60*	−1.90/3.66*	−1.95/4.33*
	Curvilinear	10.81/−3.66/0.82^#^	ns	ns
Bilirubin (mg/dl)				
	*N*	*10,854*	*10,854*	*10,021*
By Decile	Linear	1.11/0.002	1.10/0.002	1.07/0.004
	Curvilinear	ns	ns	ns
By g/day	Linear	1.11/0.003	1.11/0.002	1.07/0.01
	Curvilinear	ns	ns	ns

^a^Regression analyses examining linear and/or quadratic effect of alcohol intake conducted with the following covariates: age, race/ethnicity, current smoking status (yes/no), poverty income ratio, physical activity level (sedentary, moderate, or vigorous based on feedback on questions on activity), energy intake, and body mass index. ^b^β_0_/β_1_ for Linear Trend and β_0_/β_1_/β_2_ for Curvilinear Trend (β_0_: intercept, β_1_: linear regression coefficient, and β_2_: curvilinear regression coefficient);* Significant Linear Trend at *P* < 0.01; ^#^ Significant Curvilinear Trend at *P* < 0.01; *N* Sample Size; *ns* Non-significant Curvilinear Trend

**Table 5:** Association of liver enzyme function with alcohol consumption level (Deciles and g/day) by three different methods of alcohol intake estimation among female adults. NHANES 2001–2010^a^

		24-h recall	Usual intake by NCI method	Alcohol intake questionnaire
		Regression coefficient^b^		
Alkaline phosphatase (ALP), U/L				
	*N*	*10,387*	*10,387*	*9498*
By Decile	Linear	36.96/−0.21	38.96/−0.25	38.16/−0.54*
	Curvilinear	ns	32.91/1.73/−0.17^#^	ns
By g/day	Linear	37.31/−0.33	37.79/−0.42	37.73/−0.94*
	Curvilinear	ns	ns	41.56/−3.92/0.46^#^
Alanine aminotransferase (ALT), U/L				
	*N*	*10,359*	*10,359*	*9473*
By Decile	Linear	10.77/0.13	8.58/0.28*	10.55/0.004
	Curvilinear	ns	ns	ns
By g/day	Linear	10.57/0.19	10.09/0.35	10.05/0.27
	Curvilinear	ns	ns	ns
Aspartate aminotransferase (AST), U/L				
	*N*	*10,358*	*10,358*	*9472*
By Decile	Linear	18.10/0.34*	15.88/0.28*	16.62/0.21*
	Curvilinear	ns	ns	17.15/−0.29/0.06^#^
By g/day	Linear	17.58/0.50*	16.49/0.83*	15.78/0.88*
	Curvilinear	ns	ns	ns
Gamma glutamyl transferase (GGT), U/L				
	*N*	*10,387*	*10,387*	*9498*
By Decile	Linear	0.04/0.99*	−7.01/0.89*	−2.16/0.67*
	Curvilinear	ns	ns	−0.44/−0.96/0.19^#^
By g/day	Linear	−1.49/1.47*	−4.76/2.47*	−4.73/2.77*
	Curvilinear	ns	ns	ns
Bilirubin (mg/dl)				
	*N*	*10,382*	*10,382*	*9493*
By Decile	Linear	0.83/0.005*	0.81/0.002	0.81/0.01*
	Curvilinear	ns	0.86/−0.02/0.001^#^	ns
By g/day	Linear	0.82/0.01*	0.81/0.01	0.81/0.01*
	Curvilinear	ns	0.73/0.06/−0.01^#^	0.78/0.04/−0.004^#^

^a^Regression analyses examining linear and/or quadratic effect of alcohol intake conducted with the following covariates: age, race/ethnicity, current smoking status (yes/no), poverty income ratio, physical activity level (sedentary, moderate, or vigorous based on feedback on questions on activity), energy intake, and body mass index. ^b^β_0_/β_1_ for Linear Trend and β_0_/β_1_/β_2_ for Curvilinear Trend (β_0_: intercept, β_1_: linear regression coefficient, and β_2_: curvilinear regression coefficient); * Significant Linear Trend at *P* < 0.01; ^#^ Significant Curvilinear Trend at *P* < 0.01; *N* Sample Size; *ns* Non-significant Curvilinear Trend

There were significant effects of age (as age increased ALP decreased and AST, ALP, and GGT increased), gender (males were higher for all markers), and ethnicity (the effects varied depending on the specific marker in question) on liver markers; ALP and GGT decreased as poverty income ratio increased and there was no effect of hypertension status on liver markers (data not shown). All these effects were similar regardless of method used to assess alcohol intake. There were no significant interactions or only a few sporadic significant interactions (which may be due to chance given the large number of interactions evaluated) of alcohol intake with gender, race/ethnicity, socioeconomic status, and presence of hypertension (data not shown). However there were significant interactions of alcohol intake for all three measures of intake assessment with age for ALP and GGT. For ALP there was a positive relationship of alcohol intake up until about age 35–40 years and thereafter higher intake was associated with slightly lower ALP. Also, GGT increased as age increased (data not shown).

## Discussion

Approximately 25 % of adults consume alcohol on a given day as assessed by the 24-h recall method and ~60 % adults regularly consume alcohol as assessed by the alcohol questionnaire method. Previous studies have shown that approximately 50 % of US adults are regular drinkers [2]. An analysis of data from NHANES 2003–2006 using 24-h recall also found that about 33 % of men and 17 % of women consumed some amount of an alcoholic beverage on a given day [25]. The average regular intake of alcohol observed in this study, as assessed by either by questionnaire or the usual intake NCI method, was about 11 g/d (~16 g/d for men and 6 g/d for women) and is considered moderate intake by the Dietary Guidelines for Americans, 2010 definition [2].

### Effects of alcohol on liver function

To the best of our knowledge, this study provides the most current, detailed data on the association of liver enzymes with graded levels of alcohol intake in a very large (most likely the largest) representative sample of the U.S. population. By combining data collected by NHANES over the last 10 years on over 20,000 adults, quantitative estimates of the relationship between alcohol intake and multiple liver enzymes could be calculated with a very high level of sensitivity to graded changes in alcohol intake of the US population. This study demonstrates that even very modest levels of alcohol intake can significantly affect liver enzymes and the most sensitive measure of alcohol intake is the enzyme GGT which is potentiated by alcohol intake as low as 7–14 g/day.

Our data present both linear and curvilinear (quadratic) equations (when the latter are significant) that can be used to assess impact of alcohol on liver enzymes. The linear component of both sets of equations have the same direction, but the curvilinear equations have the quadratic element which modifies the direction and/or magnitude of change as alcohol intake increases. For example, both the linear elements of ALP (in both linear and curvilinear equations) are all negative, but the curvilinear component is positive which indicates as alcohol intake increases further the magnitude of the drop in ALP decreases. While the linear equations are useful, when curvilinear equations were significant these will provide a better fit of the relationship of alcohol intake with liver enzymes.

Excessive alcohol consumption can cause liver diseases including fatty liver, hepatitis and cirrhosis [26–28]. Since alcohol is mainly metabolized by the liver, it is a primary site of alcohol-induced adverse health effects. Alcohol consumers had significantly higher AST and GGT activities compared to non-consumers confirming previous findings demonstrating that alcohol intake is associated with increased hepatic enzyme activities [12, 13]. Changes in liver enzymes activities are biomarkers of liver damage and are routinely assessed for diagnostic purposes and as part of physical examinations [22–24]. Abnormal activities of liver enzymes are also strong predictors of mortality associated with liver disease, cardiovascular disease, diabetes and cancer [29–33]. However, the activities of liver enzymes AST and GGT in alcohol consumers in our study did not approach levels that would be considered clinically abnormal [34]. It may be possible that the changes in the activities of liver enzyme within the normal range are not benign and additional research is required to determine if they are associated with subsequent development of liver disease.

The association between alcohol consumption and GGT has previously been demonstrated and is a widely used index of excessive alcohol intake [12, 13, 35, 36]. Consistent with the literature, in our study serum GGT appears to be the most sensitive measure of alcohol consumption as assessed by 24-h recall, as well as alcohol questionnaire, with respect to the difference between alcohol consumers and non-consumers. Our results support previous findings that GGT is a more sensitive indicator of moderate levels of alcohol consumption than AST and ALT [12, 13, 16]. Elevated serum GGT has also been shown to be associated with metabolic syndrome [37, 38] and is considered to be the most sensitive indicator of liver disease [39]. Previous studies have suggested the presence of a graded dose–response relationship between alcohol intake and risk of liver disease [40–43] and that GGT induction can be initiated at low doses of alcohol intake [12, 13]. In the present study we noted a gradual increase in liver enzyme activities with increasing alcohol dose with the largest dose-dependent increase noted for GGT activity. This is in agreement with studies reporting a gradual effect of increasing dose of alcohol on liver enzyme induction [12, 13, 16].

The health effects of alcohol also vary across population groups. A negative dose response relationship between consumption of alcohol and prevalence of suboptimal health was reported in a cross sectional survey from Spain [44] while a curvilinear relationship (inverse J shaped) was observed between alcohol intake and health related quality of life in Dutch population [45]. Intoxication and liquor consumption were associated with poor mental and physical health while moderate intake were associated with better health in another cross sectional study conducted in New York State [46]. Future studies, including epidemiological investigations and clinical trials, should be conducted to investigate the relationship between intake of various levels of alcohol, multiple health outcomes and all-cause mortality.

The factors responsible for the beneficial effects of moderate intake of alcohol are uncertain, although the adverse effects of higher doses of alcohol on various organ systems have been well documented. Increased HDL, apolipoprtotein A-1 and adiponectin levels, and reduction of LDL concentration, blood pressure, coronary blood flow, platelet aggregation, fibrinogen levels and inflammation resulting from moderate alcohol intake have been suggested as mechanisms that could explain the beneficial effect of moderate alcohol intake [47, 48]. One additional mechanism that may explain some of the beneficial effects of lower doses of alcohol could be its effects on the liver. The liver is the organ primarily responsible for detoxifying a wide variety of metabolic and environmental toxins so consumption of low doses of alcohol could, by potentiation of key liver enzyme systems such as cytochrome P450, enhance its ability to remove toxic compounds from the body. It should be noted that levels of GGT increase with obesity but the effects of BMI, as well as other covariates, were adjusted for in our analyses [49].

### Methods for assessing alcohol intake

In this study we used data from 3 methods of assessing alcohol intake, and evaluated the association of these intake estimates with liver enzyme functions. It is interesting to note that, regardless of the method used to assess alcohol intake, each with their own measurement error issues, alcohol consumption significantly altered AST, ALT & GGT. In large surveillance studies of the U.S. population, 24-h recall is the preferred method of assessing of dietary intake and determining changes in consumption patterns, and these data are used in developing regulations, policies, and dietary standards. The 24-h recall method provides as estimate of an individual’s alcohol consumption on the day of recall (acute intake) but does not capture day-to-day variation in intake and appears to substantially overestimate annual alcohol consumption. The alcohol intake questionnaire used in NHANES is designed to assess long term consumption of alcohol (during the past 12 months as well as over the lifetime) but might not accurately represent actual alcohol intake since it relies on memory and estimation of the actual amount consumed by the consumer and may be difficult for subjects to report accurately. The NCI method can estimate usual intake distributions for episodically-consumed dietary components like alcohol even when there are large proportions of zero intakes on any given day. In this study we found that the NCI method and the alcohol intake questionnaire provided very similar estimates of intake with the population means varying by only 0.1 g/d and were predictive of multiple markers of liver function, the biological measures of alcohol intake we examined. Therefore, they both appear to be useful methods to estimate alcohol intake. While various methods of alcohol assessment appear to be useful in assessing the relationship with the specific physiological measures evaluated in this study, namely liver enzymes, researchers should carefully consider specific objectives of their research and the impact of measurement error of the method used and study participant burden to provide intake information before selecting an alcohol assessment approach.

### Study limitations and strengths

The limitations of our study include the inability to determine cause-effect relationship due to cross-sectional design of NHANES. Another limitation is the potential for bias in self-reported intakes since alcohol intake is often underestimated [50] and the potential for measurement error of intake instruments to influence our results. However, it should be noted that alcohol intake as estimated by the questionnaire and NCI method were quite similar and while we would expect measurement error to attenuate our ability to detect relationships, various significant relationship were still found. Other factors which may impact markers of liver function (e.g., health condition, concomitant medication use. etc.) were not considered in these analyses and therefore residual confounding may have influenced our results. A major strength of our study is the use of a large nationally representative population-based sample of adults.

## Conclusions

The data presented in this study show that moderate alcohol consumption affects liver function in a dose-dependent manner. Use of data from the NHANES survey, especially when combined over multiple years, provides an opportunity to study potential adverse effects of dietary constituents, especially those thought to be associated with changes in biomarkers routinely assessed as part of the NHANES survey. In addition, this study indicated usual intakes determined using the NCI method and an alcohol intake questionnaire yield similar estimates of alcohol intakes and relationships with liver enzymes.

## Abbreviations

NHANES, National Health and Nutrition Examination Survey; NCI, National Cancer Institute; NCHS, National Center for Health Statistics; CDC, Center for Disease Control and Prevention; ALP, alkaline phosphatase; ALT alanine aminotransferase; AST, aspartate aminotransferase; GGT, gamma glutamyl transferase; LS, least square; SE, standard errors
